# Application of acellular dermal xenografts in full-thickness skin burns

**DOI:** 10.3892/etm.2013.1114

**Published:** 2013-05-15

**Authors:** XIAODONG CHEN, XIANGSHENG FENG, JULIN XIE, SHUBIN RUAN, YAN LIN, ZEPENG LIN, RUI SHEN, FENGGANG ZHANG

**Affiliations:** 1 Department of Burns Surgery, The First People’s Hospital of Foshan, Foshan, Guangdong 528000;; 2 Department of Burns Surgery, The First Affiliated Hospital of Sun Yatsen University, Guangzhou, Guangdong 510080, P.R. China

**Keywords:** skin grafting, burns, slit-thickness skin, acellular dermal xenograft

## Abstract

The aim of this study was to explore the clinical value of the porcine acellular dermal xenograft (ADX) in combination with autologous split-thickness skin and pure autologous split-thickness skin grafting applied in deep full-thickness burns and scar wounds. A total of 30 patients with deep burns were randomly divided into experimental and control groups following escharectomy. The patients were separately treated with porcine acellular dermal xenograft (ADX) in combination with autologous split-thickness skin and pure autologous split-thickness skin graft. The wound healing was observed routinely and the scores were evaluated using Vancouver scar scale at different times following transplant surgery. The samples of cograft regions and the control group (pure transplant split-thickness skin autograft) were observed using light microscopy and electron microscopy, and the follow-up results were recorded. No conspicuous rejections on the cograft wound surface were observed. Compared with the control group, the cograft wounds were smooth, presented no scar contracture and exhibited good skin elasticity and recovery of the joint function. The cografted skin combined well and displayed a clear and continuous basal membrane, as well as gradually combined skin structure, a mature stratum corneum, downward extended rete pegs, a mainly uniform dermal collagen fiber structure, regular alignment, and fewer blood capillaries. Clear desmosome cograft regions were identified among heckle cells, as well as a clear and continuous basal membrane. The cografted skin of the combined split-thickness autograft and the acellular heterologous (porcine) dermal matrix showed an improved shape and functional recovery compared with the pure split-thickness skin autograft. The combination of the meshed ADX and the split-thickness skin autograft applied in deep full-thickness burns and scar wounds may induce tissue regeneration via dermis aiming. This method also has superior shape and functional recovery, and has an extensive clinical application value.

## Introduction

In the wound-healing process, dermal substitutes play a guiding and supporting role during the cell and blood vessel recovery process. As a dermal regeneration template, they also promote the guided regeneration function of the tissue, reduce scar hyperplasia and improve the quality of wound-healing (1). In 1995, Livesey *et al* (2) discovered the acellular dermal matrix (ADM) by using physical and chemical processes to remove allogeneic cell components from the skin. ADM combines well with the wound and promotes the ingrowth of fibroblasts on the surrounding normal tissue. After one week, new blood vessels are formed as a permanent dermal replacement. In one study (3), matrix enzyme treatment was used to obtain ADM via cyclical compression technology. Ma *et al* (4) reported that porcine ADM and an autologous split-thickness skin graft film composite were able to effectively treat full-thickness skin defects in animals and improve the quality of wound healing. Since 1997, the Foshan First People’s Hospital Burns and Plastic Surgery Unit have used xenogeneic ADM as a temporary wound-covering material to treat burn wounds and have obtained satisfactory clinical results (5). Since the satisfactory outcome of the first successful use of an acellular dermal allograft and an autologous mesh cograft by Wainwright *et al* (6,7), composite sheets comprising a combination of different dermal matrices and autologous films, including skin films and skin particles, have been widely used in clinical applications (8–10). Acellular dermal grafts have been used to repair perineal hernia (11), complex scalp defects (12) and eyelid defects (13). To identify improved methods of repairing deep-burn wounds or scar removal wounds, 30 patients with deep burns who underwent crust cutting were treated using a combination of meshed acellular dermal xenograft (ADX) and split-thickness skin autograft from January 2002 to December 2003.

## Subjects and methods

### General data

A total of 30 cases were enrolled in the present study (20 males and 10 females, aged 18–60 years) between January 2002 and December 2003. The burn area was 25–60% of the total body surface area (TBSA), with the third-degree burn area ≤40%. The smallest region of cografting was ∼0.5% and the largest was ∼3%. All patients presented thermal burns, with no exposed bones, joints, nerves or tendons, no serious heart, liver, kidney and blood system complications and no systemic infection. This study was conducted in accordance with the Declaration of Helsinki and with approval from the Ethics Committee of The First People’s Hospital of Foshan. Written informed consent was obtained from all participants. Acellular (porcine) dermis was obtained from the Institute of Qidong Medical Supplies, China.

### ADX preparation

ADX was prepared according to a previously described method (2). The crust of the deep-burn wound was cut up to the plane of the normal tissue. The scar wound was excised to the faulty adipose tissue or deep fascial plane. Following complete hemostasis, cografting was conducted on the base of the wound. A control wound area next to the cograft plot was selected for pure grafting of split-thickness skin.

### Application of ADX in deep full-thickness burn wounds

A total of 30 deep-burn patients were selected for wound crust cutting 1 week after injury. The wounds of 20 patients were cut to the deep fascia and the wounds of the remaining 10 patients to the superficial fascia. Following complete hemostasis, the patients were treated with a combination of ADX and split-thickness skin autograft transplanted in ∼1–2% of the area, while a pure graft of split-autologous epidermal skin ∼0.15–0.25 mm thick was placed on the control wound area beside the cograft plot. The cografted and controlled areas were opened after 5–14 days.

### Wound healing observation

The observation was performed by two clinical physicians to maintain consistency. Two weeks after transplantation was considered the skin graft survival observation endpoint. The area of skin graft survival was observed using the grid number method and graft survival rate was calculated using the following formula: Skin graft survival area/original graft area ×100.

### Vancouver scar hyperplasia evaluation

Scars were described using the Vancouver Scar Scale (VSS). Melanin, hardness and scar hyperplasia height were observed at 1, 3, 6 and 12 months after transplantation and were scored by the VSS (2). The scars were evaluated descriptively using the following four indices: melanin (M), height (H), vascularity (V) and pliability (P).

The score criteria were as follows: i) M: 0, scar color similar to that of normal body parts; 1, lighter color; 2, mixed color; and 3, darker color. ii) H: 0, normal; 1, <1 mm; 2, 1–2 mm; 3, 2–4 mm; and 4, >4 mm. iii) V: 0, scar skin color similar to that of normal body parts; 1, partial pink color; 2, reddish color; and 3, purple color. iv) P: 0, normal; 1, soft (deformation under the skin in the least resistance); 2, flexible (deformation under pressure); 3, hard (inflexible, moving in blocks, resistant to pressure); 4, banding (rope-like organization and scar blanches when stretched); and 5, contracture (shortening leads to permanent deformity and scar distortion). The maximum possible score was 15 points and higher scores represented more severe scarring.

### Morphological observations

With patient consent, specimens were excised from the composite graft and control areas at 2 weeks, 8 weeks, 12 weeks, and 1 year and 10 months after surgery and were pathologically assessed by a single-blind method. The specimens were divided into two groups under sterile operating conditions. One group contained the specimens for observation under a light microscope. The specimens (0.3×1.0 cm in size) were fixed in 10% formalin, dehydrated for 5 min, embedded in paraffin, sectioned in series (5 *μ*m thick) and finally stained with hematoxylin-eosin. The specimens were then observed under an optical microscope. The second group contained specimens for observation under the electron microscope. The specimens (0.2×1.0 cm) were fixed in 10 g/l osmium tetroxide, dehydrated, impregnated in an epoxy resin, embedded and cut into thinner sections (60 *μ*m thick). The specimens then underwent uranium and lead double-staining and were observed under a CM10 transmission electron microscope (Philips, Amsterdam, The Netherlands).

### Postoperative follow-up

All the wounds of patients underwent postoperative follow-up for 6 months to 2 years.

### Statistical analysis

All data were analyzed using SPSS 13.0 software (SPSS, Inc., Chicago, IL, USA). Results were compared using a t-test. P<0.05 was considered to indicate a statistically significant difference.

## Results

### Wound-healing process and changes in appearance and function

**Figure 1.**
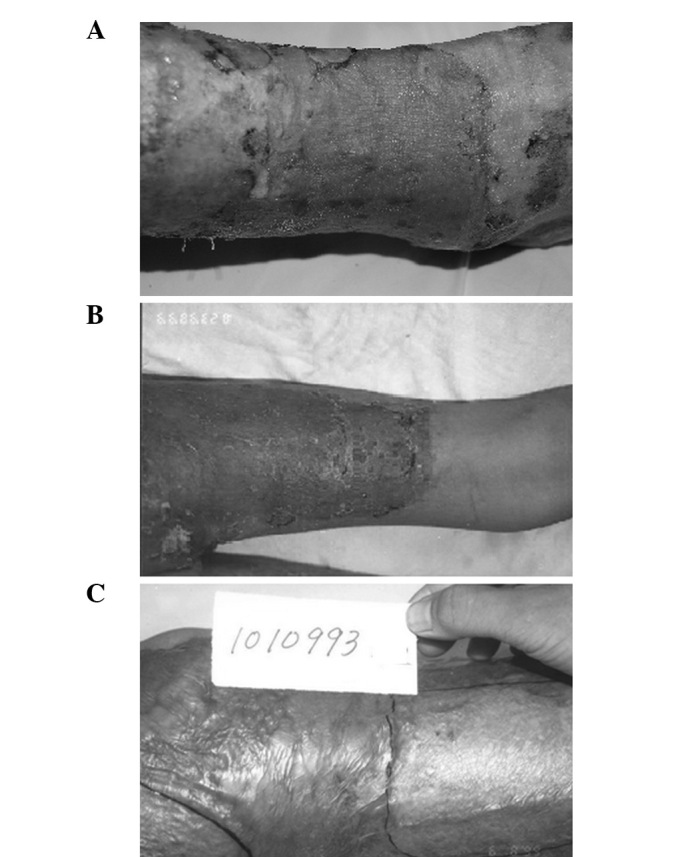
Grafts (A) 10 days and (B) 30 days after composite acellular dermal matrix (ADM) and thin split-thickness skin autograft grafting surgery. (C) The surface of the composite graft area at 90 days was smooth, with thick skin, no scar contracture and good function, was pale yellow-white in appearance and had traits similar to those of the full-thickness skin graft.

At 5 days after cograft surgery, which used a combination of meshed ADX and split-thickness skin autograft, the wounds, which were slightly dark red in color, were opened using an autologous skin graft survival method. The meshed dermal xenograft was indistinct throughout the split-autologous skin grafts. The unstable combination of the skin grafts and the base was felt when the wound was touched. At 10 days after surgery, light-colored thick-layer scalings were observed on the cografted wound. The dermal xenograft was barely visible under the autologous epidermal skin graft and the wound achieved a stabilized composite graft survival based on touch (Fig. 1A). Two weeks after transplantation, the survival rates of the composite transplant wounds and simple autologous split-thickness transplant wounds were 100%. The autologous skin graft in the control area exhibited a small amount of skin scaling, with a color similar to that of the cograft area observed 10 days after surgery. At 30 days after cografting, the composite skin was soft, smooth and thick and exhibited no shrinkage or wear, and an even interface with the flat edge of the normal skin (Fig. 1B). The autologous epidermal skin graft on the control area started to exhibit varying degrees of thick, wrinkled and hard-textured mild scar uplift. After 45 days, the cografted skin was shiny, remained soft and smooth and was slightly redder than normal; however, it was lighter in color compared with that of the control area. After 90 days of general observation, the skin on the cografted area was even and smooth. The thick and hard-wearing epidermis demonstrated no scar contracture, was pale yellow-white, exhibited good function and had traits similar to those of the full-thickness skin graft at the time of excision (Fig. 1C). The control wound exhibited surface shrinkage and elevation, and hardened with the formation of dark red contractures and a conspicuous hyperplasia scar.

### Vancouver scar hyperplasia evaluation

VSS at 1 month after burn wound healing demonstrated no significant differences between the experimental area and control area (P>0.05), while the difference was statistically significantly after 3, 6 and 12 months (P<0.05; Table I).

### Morphological observation

**Figure 2.**
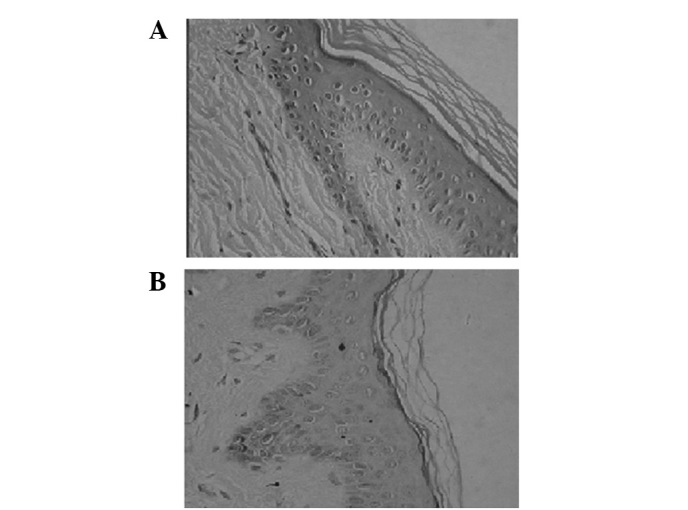
(A) Two weeks after cografting, thick desquamation, active acanthocyte proliferation and an increased number of inflamed cells and fibroblasts between the dermal template and the raw surface juncture were observed (B) 12 weeks after cografting, the cograft skin mixed together well, the basal membrane was clear and continuous, the stratum corneum was mature, rete pegs extended downward, dermal collagen fibers had a uniform structure and there were fewer blood capillaries; however, there were no appendages of the skin. Hematoxylin and eosin staining; magnification, ×100.

**Figure 3.**
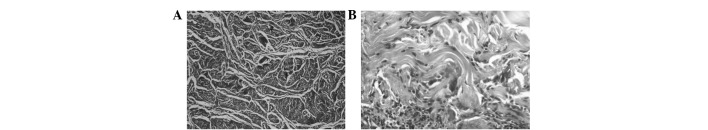
(A) Masson’s staining revealed a more regular arrangement of dermal collagen in the cograft, which was uniformly stained (magnification, ×100); (B) there was a large number of dermal fibroblasts and collagen deposition in the control group, accumulated in a disorderly manner (magnification, ×100).

**Figure 4.**
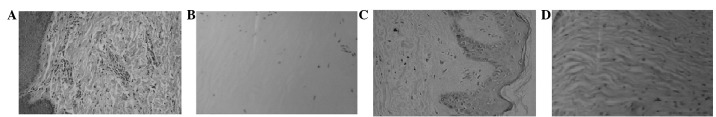
(A) Six months after cograft surgery, the dermis composition had integrity, inflammatory cells were reduced and collagen was arranged regularly; (B) One year after cograft surgery, the skin structure appeared similar to normal skin under a light microscope; (C) 1 year and 10 months after cograft surgery, the cograft skin was similar to normal skin; (D) 1 year and 10 months after graft surgery, collagen was disordered and the density was variable in the control group. Hematoxylin and eosin staining; magnification, ×100.

Two weeks after transplantation and with patient consent, three biopsy specimens were randomly selected and observed under a light microscope using a scaled layer of the thick cografted prickle cell hyperplasic area. Numerous inflammatory cells and fibroblasts in the junction of the dermal xenograft and the base of the wound were observed. The autologous epidermis, dermal xenograft and adipose layers closely interweaved with one another and were hardly separated (Fig. 2A). Under the scale-like epithelium, rich capillaries and fiber mother cells, lymphocytes, neutral white blood cells and multinucleated cells were clearly observed. However, no skin appendages were observed. Twelve weeks after cograft surgery, the skin structures had combined. The corneous layer was continuous, mature and presented downward-stretching rete pegs and dermal collagen fibers with similar structural thickness and a regular arrangement. The number of capillaries was lower than before and no skin appendages were observed (Fig. 2B). Masson’s staining revealed a more regular arrangement of the dermal collagen in the cograft, which was uniformly stained (Fig. 3A), while there was a large number of dermal fibroblasts and collagen deposition in the control area, accumulated in a disorderly manner (Fig. 3B). Six months after cograft surgery, the dermal composition had integrity, inflammatory cells were reduced and collagen was arranged regularly (Fig. 4A). Approximately 1 year and 10 months after cografting, the skin structure appeared similar to normal skin under a light microscope (Fig. 4B and C), while in the control area, collagen was disordered and the density was variable (Fig. 4D). Eight weeks after transplantation, electron microscopy examinations revealed a clear spike of desmosomes between cells, the basal membrane and hemidesmosomes. However, the basal membrane of the control area appeared fuzzy and discontinuous.

### Postoperative follow-up

Patients were followed up for 6 months to 2 years after surgery. The appearance and functional recovery of the composite transplant wound were better than those of the simple autologous split-thickness skin transplant. The survival, color, elasticity, thickness and mobility of the transplanted skin area were good. Patients treated with the composite acellular (porcine) and autologous split-thickness skin graft presented good expected results.

### Adverse reactions

No significant adverse reactions were observed during the treatment with the two types of graft, including skin ulcers or scar hyperplasia at the donor sites.

## Discussion

In patients with extensive deep burns, the use of the patient’s own skin to treat the wounds is recommended, using the principles and techniques of plastic surgery, depending on the skin source and the skin conditions. This treatment has great significance in the reduction of late surgeries, as well as in the recovery of the function and appearance of burns or scars (14). However, when deep-burn wounds and scar-excision wounds, particularly extensive wounds, are treated, the use of the patient’s own thick-skinned or autologous full-thickness skin for repair is challenging. For a timely and effective wound closure, several methods, including split-thickness skin autografting, autologous meshed skin grafting and autologous particle grafting, are currently available (9). Although these methods are effective to a certain degree, they have also demonstrated varying degrees of postoperative scar formation and dysfunction due to the lack of adequate dermal components. Balasubramani *et al* (15) considered such a lack of dermal structure or dermal components as the cause of overexpression of fibroblast cells in the wound-healing process, eventually resulting in conspicuous scar hyperplasia. Such a phenomenon may be significantly changed with dermal component (dermal structure) or dermal substitute replenishment, which greatly improves the quality of wound-healing. Hence, ADM was developed and applied to dermal wounds (2). The authors reported that dermal substitution guided the tissue regeneration. A dermal substitute may be used as a dermal regeneration template to support the infiltration of host fibroblasts, neovascularization and epithelialization during the wound-healing process, thereby reducing the scar and improving the quality of wound-healing (16). Certain scholars (17) identified that adding hyaluronic acid to porcine ADM promotes the expression of collagen types I and III and reduces the ratio of collagen type I to type III. Porcine ADM was also shown to be conducive to wound-healing, skin graft reconstruction of the basement membrane and reduction of shrinkage. Xu *et al* (18) grafted porcine ADM onto a simian rotator cuff and observed no hypersensitivity. Moreover, the skin was safely repaired and the rotator cuff of the human structure was strengthened. The effects of using human ADM and non-crosslinked porcine ADM were compared in a study concerning the repair of porcine abdominal hernia (19). The results demonstrated that vascular tissues and cells exhibited infiltration in four weeks and the muscle fascia and bioprosthesis interface had similar strengths; however, the human ADM presented a greater amount of cell and blood vessel infiltration. An animal study (20) revealed that, compared with pure autologous skin, vascular cell adhesion molecule 1 on the cograft exhibited high expression levels of latency, suggesting that the expression of vascular cell adhesion molecule 1 is significantly related to angiogenesis and the remnants of the composite skin. This result also suggested that the expression levels of vascular cell adhesion molecule 1 in the homologous and heterologous ADM cografts are different.

The structure of porcine skin is similar to that of human skin and its composition and collagen content are also similar to those of human skin grafts. Porcine collagen adhesion, hemostasis function and pain reduction traits are the same as those of humans. Hence, porcine skin as a temporary covering for wounds is widely used in clinical studies (21–23). Since 1997, we have used porcine cells in derma-clinical applications and have identified that the cograft survival time is not significantly different from that of the pure split-thickness skin autograft. At 5 days after surgery and after opening the cograft area wound, the split-thickness skin autograft was living tissue; however, the combination with the base was not stable. At this time, the fibrin exudate of the base infiltrated the bottom of the epidermis through the ADX mesh. The fibrin exudate nourished the epidermis and established a blood supply with the epidermis. At 10 days after surgery, the split-thickness skin autograft gradually blended with the stent (xenograft), which initially completed the healing process of the composite graft. The tissue sectioning confirms that two weeks after cografting, the autologous epidermis, dermis and adipose layers via cografting generally had more closely connected skin bonding that was difficult to separate. Approximately 12 weeks after cografting, the composite skin graft was soft, smooth and easy to lift. Under light microscopic observation, the skin structure integrated into one continuous and mature stratum corneum with stretched spikes. The thickness of the collagen fibers in the dermis was essentially the same and had a relatively regular arrangement. Masson’s staining revealed a generally regular arrangement of the dermal collagen, which was uniformly stained. These results suggest that the three-dimensional structure of the dermal tissue plays a guiding role in cell repair. The structure not only induces cell repair ingrowth, but also adjusts the function of repair cells to improve the mechanical state of the wound. The integrity and continuity of the dermal tissue is a necessary prerequisite in fully executing the guiding role in cell repair. The loss of dermal tissue integrity and continuity following injury, which induces the lack of guiding function, may be one of the important mechanisms affecting cell repair function and may result in cicatrix formation. The clinical application results of the 30 cases in the present study show no conspicuous rejections of the cograft wound surface after cografting with ADX and the split-thickness skin autograft, which had a high survival rate and produced a satisfactory grafting form and function. This type of cografting saves the patient’s autologous skin source and skin donor area without leaving any scars. The price of acellular dermal xenograft in China is 127 USD/10×10 cm, while the price of Integra™ in China is 3,500 USD/10×10 cm. There are no significant differences of clinical efficacy between the two methods. In conclusion, the cografting of ADX and split-thickness skin autograft is an ideal treatment method for the repair of deep full-thickness burns and scar wounds and has extensive clinical applications.

## Figures and Tables

**Table I.** Comparison of the Vancouver Scar Scale score between the experimental and control areas.

	Time point			
Group	1 month	3 months	6 months	12 months
Experimental	8.35±1.16	8.98±1.48	7.48±1.61	6.35±1.03
Control	8.98±1.22	10.35±1.68	12.23±1.24	10.54±1.75
P-value	P>0.05	P<0.05	P<0.05	P<0.05

Values are mean ± SD.
